# Circulating Tumor Cells in Soft Tissue Sarcoma: Current Evidence and Clinical Implications

**DOI:** 10.3390/cancers18101542

**Published:** 2026-05-10

**Authors:** Carolina Mendez-Guerra, Jose Chacon, Irvin E. Altamirano, W. Rodrigo Calmet Rocca, Juan Pretell-Mazzini

**Affiliations:** 1 Facultad de Ciencias de la Salud, Universidad Peruana de Ciencias Aplicadas, Lima 15067, Peru; pcmecarm@upc.edu.pe; 2 School of Medicine, American University of Integrative Sciences, Christ Church, Bridgetown BB11114, Barbados; jchacon@auis.edu; 3 Facultad de Medicina, Universidad Peruana Cayetano Heredia, Lima 15102, Peru; irvin.altamirano@upch.pe; 4 Department of Surgery, Beth Israel Deaconess Medical Center, Harvard Medical School, Boston, MA 02115, USA; wcalmetr@bidmc.harvard.edu; 5 Division of Orthopedic Oncology, Miami Cancer Institute, Baptist Health System South Florida, Plantation, FL 33324, USA; 6 Department of Orthopedic Surgery, Herbert Wertheim College of Medicine, Florida International University, Miami, FL 33199, USA

**Keywords:** soft tissue sarcoma, liquid biopsy, circulating tumor cells, precision oncology

## Abstract

Soft tissue sarcomas (STS) comprise a rare and highly heterogeneous group of mesenchymal-derived malignancies, accounting for less than 1% of all cancers and characterized by diverse histologic and molecular subtypes. Despite their low incidence, STS account for a disproportionate burden of cancer-related morbidity and mortality, largely driven by their risk of metastatic dissemination. Early detection of metastatic spread is a cornerstone of preoperative staging, treatment planning, and postoperative monitoring in patients with STS. Although conventional imaging modalities remain fundamental for surveillance of metastatic disease, they may fail to accurately detect metastatic sites and provide limited insight into tumor biology. Advances in precision medicine have positioned liquid biopsy as a minimally invasive approach for the analysis of tumor-derived material, facilitating characterization of tumor biology and identification of prognostic biomarkers. Circulating tumor cells (CTCs) represent intact and viable tumor cells that provide unique genomic and phenotypic traits that could not be assessed using acellular tumor-derived material. They have emerged as promising biomarkers for monitoring disease progression, assessing treatment response, and stratifying prognosis. Particularly, their clinical value as prognostic biomarkers has been established in epithelial-derived malignancies. Despite these advances, the role of CTCs in STS remains largely investigational, mainly due to STS heterogeneity and the lack of standardized protocols for detection across platforms. Therefore, this narrative review summarizes the biomolecular mechanisms underlying CTCs in STS, including the role of phenotypic plasticity in tumor intravasation, anoikis resistance and its interaction with the tumor microenvironment, and stem cell-like phenotypes in tumor initiation at distant sites. Furthermore, we discuss current methodologies for CTC detection, highlighting emerging approaches developed to address the limitations of conventional methods. Finally, we provide a critical overview of subtype-specific detection strategies, as well as their clinical implications in treatment response monitoring and prognostic assessment.

## Simple Summary

Soft tissue sarcomas comprise a rare and heterogeneous group of cancers of soft tissues characterized by their high ability to spread to other parts of the body. Early detection of this spread is crucial for preoperative staging, treatment planning, and postoperative monitoring. Conventional imaging modalities have important limitations; particularly, they fail to characterize tumor biology. In that instance, liquid biopsy emerges as a minimally invasive approach that analyzes tumor-related material in the blood. Circulating tumor cells represent intact and viable tumor cells that travel through the bloodstream and can provide real-time information about disease progression as well as monitor therapy response over the course of treatment. Although its use has been established in epithelial-derived malignancies, their clinical utility in soft tissue sarcomas remains under investigation. This review summarizes current knowledge on circulating tumor cells in soft tissue sarcomas while discussing their potential use for disease monitoring and predicting survival outcomes.

## 1. Introduction

Soft tissue sarcomas (STS) comprise a rare and highly heterogeneous group of mesenchymal-derived malignancies, representing less than 1% of all cancers [1,2]. These tumors encompass more than 50 distinct histologic subtypes and can be broadly classified, based on their genetic profiling, into: (i) sarcomas with specific genetic alterations, including translocation-related sarcomas (TRSs); and (ii) complex karyotype sarcomas (CKSs) [1,3]. STS account for a disproportionate burden of cancer-related morbidity and mortality, with approximately 13,590 new cases and 5200 deaths occurring annually in the United States alone [4]. Despite the majority of STS patients being diagnosed at a non-metastatic stage, with approximately 10% presenting with synchronous metastases [5], it has been estimated that 40 to 50% will ultimately develop metastasis [6]. In this context, recent reports have shown that, although overall survival in metastatic STS has improved over the last two decades [5], pulmonary metastases continue to be the leading cause of death, further highlighting the importance of early detection of metastatic dissemination [7].

Early detection of metastatic spread is a cornerstone of preoperative staging, treatment planning, and postoperative monitoring. Traditionally, chest imaging has been fundamental for the surveillance of pulmonary metastases, as these represent the most common site of distant spread, with approximately 20% of patients ultimately developing pulmonary metastases [6]. Nonetheless, conventional imaging may fail to accurately detect metastatic sites and to provide optimal insight into the histologic subtype and biological behavior of STS [8]. Furthermore, delayed relapse detection, limited reproducibility due to inter- and intra-observer variability and differences in imaging protocols, and poor real-time disease monitoring have prompted the development of liquid biopsy techniques to better characterize metastatic disease [9,10].

Liquid biopsy comprises various analyses of tumor-derived material released into the bloodstream, including circulating tumor cells (CTCs), cell-free DNA (cfDNA), circulating tumor DNA (ctDNA), and microRNAs. This minimally invasive approach has transformed the landscape of tumor biology by enabling dynamic molecular characterization and improving the understanding of longitudinal intratumoral heterogeneity [11]. CTCs represent intact and viable tumor cells that provide unique features that cannot be obtained through acellular biomarkers, such as genomic and transcriptomic information as well as functional and phenotypic traits [12]. The value of CTC detection for predicting disease progression in epithelial-derived cancers has been well established. CTC detection has been shown to be a reliable prognostic biomarker in early-stage and metastatic breast cancer and has also been regarded as a promising biomarker for clinical decision-making in prostate and colorectal cancer [13,14,15]. Despite these advances, the clinical utility of CTCs in STS remains under investigation, and the mechanisms underlying their generation and implications in cancer progression require further clarification.

Therefore, the aim of this review is to summarize the mechanisms underlying the generation of CTCs in STS and their role in the metastatic cascade, critically evaluate the currently available detection methodologies, and examine their clinical implications in disease-progression surveillance, treatment-response monitoring, and prognostication. This review included pediatric, adolescent and young adult, and adult STS studies. Mixed sarcoma studies were only considered for contextualization when directly relevant to CTC research in STS.

## 2. Mechanism of Progression

The metastatic cascade is a tightly regulated, multistep process in which cancer cells undergo extensive metabolic network rewiring that enables intravasation into the bloodstream, survival under hostile microenvironments, and ultimately the establishment of distant metastatic lesions [16]. Initially, cancer cells must acquire invasive and migratory capacities that facilitate intravasation, a process frequently associated with epithelial–to-mesenchymal transition (EMT) [17]. Once in circulation, tumor cells must survive highly inhospitable conditions, primarily through activation of molecular pathways that promote resistance to apoptosis following detachment from the extracellular matrix, a phenomenon known as anoikis resistance [18]. Finally, successful metastatic colonization requires cancer cells with tumor-initiating capacity to establish growth at distant sites, a process that frequently involves immune modulation of the tumor microenvironment (TME) [19]. Notably, although millions of cancer cells may shed into the bloodstream, only about 0.01% are ultimately capable of successfully metastasizing [20]. Tumor cell dormancy among disseminated tumor cells (DTCs) is essential in this process, as it plays an important role in facilitating immune cell shielding and persistence at distant sites [21].

**Figure 1:**
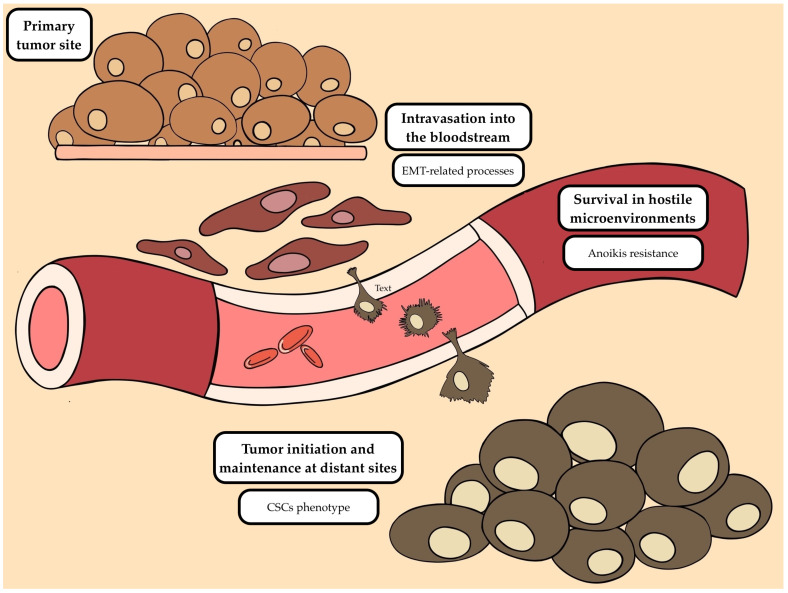
Schematic representation of the metastatic cascade. Tumor cells originating from the primary tumor site undergo epithelial-to-mesenchymal transition (EMT)-related processes to facilitate intravasation into the bloodstream. Circulating tumor cells (CTCs) must survive in hostile microenvironments through the activation of several mechanisms, including resistance to anoikis. Subsequently, they extravasate at distant sites, leading to tumor initiation and maintenance, partly driven by cancer stem cell (CSC) phenotypes.

In the following paragraphs, we will address the role of phenotypic plasticity, anoikis resistance and the TME, and cancer stem cell-like phenotypes in CTCs (Figure 1).

### 2.1. Phenotypic Plasticity

Phenotypic plasticity refers to a dynamic, reversible process in which cancer cells switch between cellular states to facilitate metastatic spread. This phenomenon arises from cell-intrinsic factors, including genetic mutations, epigenetic modifications, and chromosomal instability, as well as cell-extrinsic environmental cues that modulate cellular reprogramming [22]. Cancer cell plasticity includes EMT as a core mechanism, as well as other important processes such as cancer stemness and the awakening of dormant DTCs [23]. EMT is a process in which cancer cells acquire mesenchymal-associated features to increase their invasive and migratory capacities, while losing epithelial traits [24]. It is characterized by downregulation of epithelial cell markers, cell polarity disassembly, disruption of intercellular junctions, and loss of basement membrane adhesion molecules [25]. Although EMT has been predominantly studied in epithelial-derived malignancies, emerging evidence suggests that, owing to the dynamic nature of EMT, certain mesenchymal-derived malignancies may reside in an intermediate “metastable” state enabling them to undergo EMT-related processes [17].

Importantly, distinguishing between the molecular reprogramming an epithelial cell experiences to attain a fully mesenchymal phenotype and the EMT-related processes that mesenchymal tumor cells may undergo during the metastatic cascade is important. In carcinomas, both EMT and mesenchymal-to-epithelial transition (MET) are driven by a variety of EMT-related transcription factors, such as Snail, Slug, TWIST1, and ZEB1/2, promoting phenotypic changes that ultimately enhance invasiveness and migratory capacity [17]. In contrast, as outlined earlier, mesenchymal-derived malignancies, such as STS, may exist in an intermediate “metastable” phenotype between epithelial and mesenchymal states that allows them to undergo EMT-related processes under certain conditions [17]. In this context, these processes do not represent a true epithelial-to-mesenchymal transition, but rather partial and reversible shifts in cellular phenotype within an already mesenchymal lineage. Evidence has evaluated the transcriptional regulatory networks involved in this process. Preclinical studies in rhabdomyosarcoma (RMS) have demonstrated that signaling pathways such as Sonic Hedgehog/GLI and AKT/mTOR may regulate EMT-related processes [26]. Similarly, studies in chondrosarcoma have shown that CCL21/CCR7 and SDF-1/CXCR4 signaling can activate ERK/MEK and PI3K/AKT pathways, upregulating EMT-related regulators such as Slug and Snail and promoting phenotypic changes, including E- to N-cadherin switching [27,28]. Collectively, these findings support the concept that EMT is a dynamic process that mesenchymal malignancies may partially undergo, and that this overlaps with those described in epithelial malignancies.

EMT-related processes may be particularly important in malignancies displaying both epithelial and mesenchymal features. Synovial sarcomas (SS), given their biphenotypic morphology, have been the focus of recent investigations. An in vitro study proposed that the expression of matrix metalloproteinase-14 (MMP-14) may enhance SS invasion and migration by triggering EMT-related processes [29]. It was shown that MMP-14 expression in SS correlates negatively with E-cadherin and positively with N-cadherin and vimentin [29]. Moreover, MMP-14 expression has been found to be higher in stages III and IV compared with stages I and II, which further supports its role in distant colonization [29]. Additionally, the role of epidermal growth factor receptor (EGFR) in promoting migration and survival via EMT-associated signaling has also been investigated. EGFR-mediated activation of TGF-β, Snail, and AKT, key regulators of EMT, has been shown to enhance CTC motility [30]. Furthermore, EGFR signaling has also been implicated in tumor cell dormancy, facilitating the survival of DTCs in the bloodstream [30]. Consistently, prognostic studies have found significantly worse survival outcomes in patients with negative E-cadherin expression and overexpression of Snail and TGF-β1, supporting the role of EMT-associated pathways in sarcoma progression [31,32].

### 2.2. Anoikis Resistance and Tumor Microenvironment

Under physiological conditions, epithelial cells undergo programmed cell death upon detachment from the extracellular matrix, a process known as anoikis [33]. Anoikis resistance, largely mediated by anoikis-related genes (ARGs), plays a pivotal role in facilitating cancer cell survival in the bloodstream and in modulating the TME. The landscape of ARGs in STS has only been recently explored through computational transcriptomic analyses identifying potential prognostic biomarkers and patterns of immune cell infiltration within the TME. ARG-related scoring systems have been shown to efficiently predict survival and immunotherapy response, supporting their potential clinical utility [34]. Additionally, the role of *CASP8* and FADD-like apoptosis regulator (*CFLAR*) in STS represents a potential therapeutic target due to its role in apoptosis and inflammation. Higher survival rates have been found in patients classified in the high-*CFLAR* expression group, establishing it as an independent prognostic factor in STS [35]. *CFLAR* expression has also been found to positively correlate with CD8^+^ T cells, M1 macrophages, and monocytes, which further supports its role in immune modulation of the TME in STS [35]. Of note, several molecular processes involved in TME modulation of STS are currently under investigation, including those related to long noncoding RNA and pyroptosis [36,37,38]. However, these will not be further discussed as they lie beyond the scope of this review.

### 2.3. Cancer Stem Cell Phenotype

Tumor initiation and maintenance at distant locations are highly dependent on the tumor-initiating capacity of metastatic cancer cells, a property often attributed to cancer stem cell-like phenotypes. Cancer stem cells (CSCs) are characterized by their self-renewal and tumor-initiating capacity, and by their ability to differentiate into more specialized progeny [39]. They represent a small subpopulation of tumor cells that play an important role in tumor progression, chemoresistance, recurrence, and metastasis [40]. Although the role of pluripotency-associated genes has been predominantly investigated in bone sarcomas [41], recent findings are beginning to elucidate their role in STS. Cancer stem cell-like features have been reported in a subset of embryonal RMS cells [42,43,44,45], whereas most tumor cells in *PAX3–FOXO1*–positive alveolar RMS have been proposed to behave biologically like stem cells, characterizing this subtype as a stemness-driven disease [46]. Additionally, the activation of key regulators of stem cell properties in SS appears to be controlled by the *SYT–SSX* fusion gene [47]. A subpopulation of SS tumor cells has been reported to exhibit high levels of pluripotency factors such as Sox2, Oct4, and Nanog, demonstrating in vitro self-renewal capacity and in vivo tumorigenicity [48]. These findings suggest that the role of CSC phenotypes in CTCs is highly dependent on sarcoma subtype and even on molecular phenotypes. Further investigation into the role of CSCs in STS is warranted, as the CSC theory proposes that, despite the vast molecular and phenotypic heterogeneity of STS, common biological features may exist that could be identified for future therapeutic targeting [46].

## 3. Methodologies for Detection of CTCs

### 3.1. Liquid Biopsy: Overview, Advantages, and Limitations

The application of liquid biopsy for the detection of tumor-derived material has emerged as a promising approach for isolating and analyzing tumor components from bodily fluids, such as blood, plasma, urine, and cerebrospinal fluid [9]. Its fundamental advantage lies in enabling the characterization of tumor biology and dynamics while using minimally invasive techniques for sampling. This approach is particularly important for the characterization of tumors located near critical neurovascular structures, as their surgically challenging locations typically limit adequate management. Another aspect worth highlighting is its capacity to detect multiple tumor components shed simultaneously from distinct metastatic sites, a phenomenon that may be missed by single-site tissue sampling. Additionally, liquid biopsy is reproducible, as repeated blood samples are highly feasible and well-tolerated by patients, and allows for real-time monitoring of tumor dynamics over the course of treatment, enabling a longitudinal characterization [9,49]. Despite these advantages, CTC isolation and detection remain technically demanding largely due to the low abundance of tumor material shed into circulation, with fewer than one CTC per milliliter of blood among millions of leukocytes [50]. Similarly, their isolation and detection require specialized equipment and advanced molecular testing [51]. Moreover, the lack of standardized protocols across platforms introduces inter-laboratory variability, which limits reproducibility and clinical translation [9]. In the context of STS, liquid biopsy has emerged as an innovative tool for disease characterization and, although under investigation, it has laid the foundation for the development and adaptation of CTC detection strategies applicable to these diseases [8].

### 3.2. Conventional Methodologies for CTC Detection in STS

Several conventional methodologies for CTC detection in STS have been reported in the current literature, each relying on specific operational principles. Reverse transcription polymerase chain reaction (RT-PCR) has been proven to be particularly useful in detecting tumor-specific fusion gene transcripts in patients with TRSs. For example, RT-PCR targeting the *ASPSCR1-TFE3* fusion transcript has been used to monitor CTCs in peripheral blood samples of patients with alveolar soft part sarcoma [52]. Despite its advantages, RT-PCR requires a known molecular target for identification of the relevant fusion gene transcript, which may not be readily available at initial diagnosis, making its applicability largely limited to TRSs [53]. Notably, advanced PCR-based techniques, such as droplet digital PCR (ddPCR), have been developed to provide higher sensitivity and quantitative detection of scarce fusion transcripts; however, their use remains limited to TRSs, as they remain dependent on the identification of a known molecular target. Moreover, CTC separation prior to RT-PCR may improve detection specificity, as whole blood samples contain cellular and molecular components that may generate RT-PCR signals and ultimately compromise the accuracy of CTC detection. Among the reviewed studies, CTC isolation or enrichment was not consistently performed, with the majority of studies using whole blood or cellular blood fractions without CTC-specific enrichment [52,54,55], while one study described the use of Ficoll–Hypaque density gradient centrifugation for the enrichment of mononuclear cells [56].

**Figure 2:**
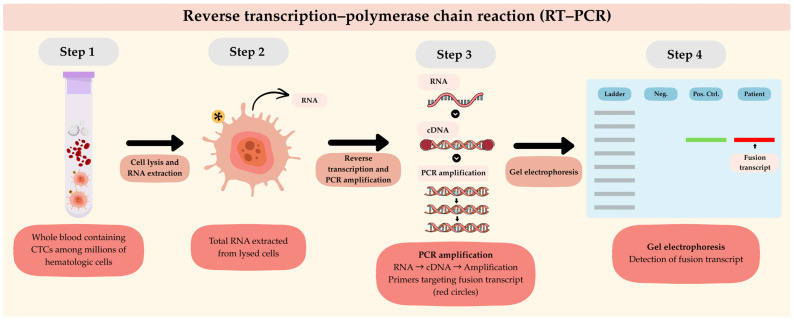
Schematic representation of reverse transcription–polymerase chain reaction (RT-PCR) for the detection of fusion transcripts in circulating tumor cells (CTCs). Whole blood samples containing CTCs undergo cell lysis and RNA extraction, followed by reverse transcription of RNA into complementary DNA (cDNA) and subsequent PCR amplification. The amplified products are then analyzed by gel electrophoresis to confirm the presence of fusion transcripts, with the red band indicating a positive patient sample and the green band indicating the positive control. The yellow star indicates CTCs.

**Figure 3:**
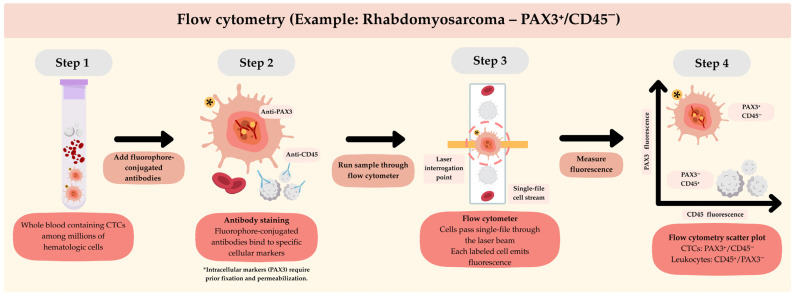
Schematic representation of flow cytometry for the detection of circulating tumor cells (CTCs), using PAX3^+^/CD45^−^ rhabdomyosarcoma tumor cells. Fluorophore-conjugated antibodies target specific cellular markers, including PAX3 (nuclear transcription factor). Cells are then analyzed individually by flow cytometry, where fluorescence signals are measured. Rhabdomyosarcoma CTCs are identified based on a PAX3^+^/CD45^−^ phenotype. The yellow star indicates CTCs.

**Figure 4:**
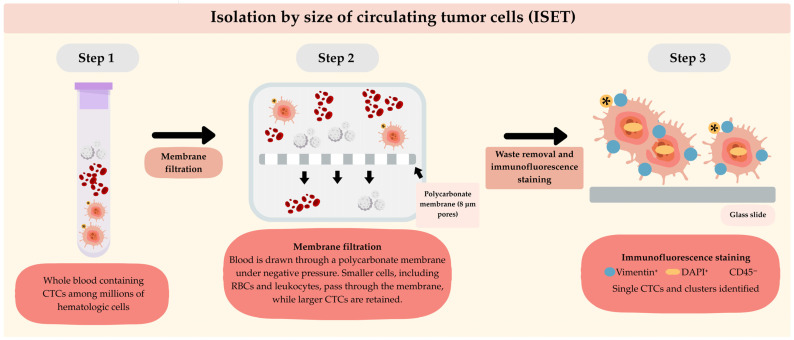
Schematic representation of isolation by size of circulating tumor cells (ISET). Whole blood samples undergo membrane filtration through a polycarbonate membrane with defined pore size, allowing smaller cells, including red blood cells (RBCs) and leukocytes, to pass while retaining larger circulating tumor cells (CTCs). Subsequently, retained cells are processed with immunofluorescence staining. The yellow star indicates CTCs.

The use of flow cytometry for CTC detection in STS has also been reported. This technique relies on the expression of surface biomarkers for the immunophenotypic characterization of tumor cells. Nevertheless, the lack of specific biomarkers per sarcoma subtype, their high molecular and histological heterogeneity, and the inability to capture CTC clusters due to single-cell gating constitute significant limitations [8]. Alternatively, isolation by size of epithelial tumor cells (ISET) offers a marker-independent method for CTC detection. This technique employs polycarbonate membrane filtration to capture CTCs based on their size relative to leukocytes. This method was tested in eleven patients with metastatic STS, in all of whom CTCs were detected at a rate of 2 to 48 cells per 8 mL of blood [57]. The use of immunohistochemistry following capture of CTCs by ISET provides additional morphological and protein-level characterization, but this technique is marker-dependent, which, as described earlier, constitutes an important limitation. It is important to highlight that the performance parameters of these methods are not fully validated and remain inconsistent across studies, largely due to the low number of CTCs in circulation and STS tumor heterogeneity [53]. Please refer to Figure 2, Figure 3 and Figure 4 for the schematic representation of the conventional methods used for CTC detection.

### 3.3. Novel Techniques for CTC Detection in STS

In light of the aforementioned, novel techniques for CTC detection have been proposed to address the limitations of conventional techniques. The CellSieve System (Creatv MicroTech, Rockville, MD, USA) initially captures CTCs by size exclusion using a size-based low-pressure microfiltration system. This is followed by morphological assessment and immunofluorescent marker staining of captured CTCs [58]. It is important to highlight that this technique surpasses the need for sarcoma-specific surface antigens, which are a significant limitation of mesenchymal-derived malignancies, and requires minimal cell processing that preserves cellular biology for further characterization. This technique was validated in a cohort of patients with multiple STS subtypes, in which both single CTCs and CTC clusters were identified in 35 of 54 samples [59].

Moreover, microfluidic technologies have emerged as promising platforms for advancing single-cell-based cancer research, as they allow the characterization of intratumoral heterogeneity and dynamic interactions within the TME [60]. The On-chip Sort microfluidic chip-type cell sorter (On-chip Biotechnologies, Tokyo, Japan) is a technique that exploits differences in size and deformability between CTCs and normal blood components for tumor cell isolation, without the need for labeling with antibodies or any other marker [61]. Additionally, it integrates flow cytometry using the FISHMAN-R system (On-chip Biotechnologies, Tokyo, Japan) and, unlike other devices, enables repeated sorting cycles without inducing cellular damage. In this regard, CTCs were successfully isolated from 10 mL of whole blood in a patient with locally advanced myxofibrosarcoma, and the tumor-specific mutation was subsequently confirmed in the recovered cells [62]. Another technique recently introduced by the CIRCUS pilot study is a combined approach using Parsortix (ANGLE plc, Guildford, UK), a microfluidic enrichment system that captures CTCs through a stepped chamber based on size and deformability, and DEPArray (Menarini Silicon Biosystems, Bologna, Italy), which isolates individual cells using electric fields for downstream single-cell analysis. This combined approach was validated in 13 STS patients, demonstrating CTC isolation feasibility [63]. Collectively, even though these studies establish the technical feasibility of novel platforms in STS, large-scale prospective validation studies must be conducted before clinical utility in STS is fully established.

Interestingly, broader oncology research has recently focused on the development and applicability of patient-derived models, such as patient-derived organoids and CTC cultures, as well as single-cell multi-omics. These cutting-edge technologies represent powerful tools for deeply characterizing both intra- and intertumor heterogeneity, as well as providing insight into disease progression and treatment response [64,65]. In this regard, a recently published study established 44 soft tissue and bone sarcoma organoids that recapitulated the histological features, cellular diversity, and genetic profiles, including mutations, of their corresponding tumors [66]. In addition, single-cell multi-omics profiling in desmoplastic small round cell tumors and epithelioid sarcoma has provided further insight into tumor heterogeneity, cellular plasticity, and clinically relevant molecular signatures [67,68].

### 3.4. Implications of CTC Detection Methodologies Across Sarcoma Subtypes and in Early Dissemination

Another important aspect to consider is the method used for CTC detection, as negative or less favorable results may reflect limitations of the detection technique rather than differences in tumor biology across sarcoma subtypes. For instance, although minimal residual disease (MRD) has been detected using RT-PCR in bone marrow samples of patients with alveolar RMS, less favorable results have been found in peripheral blood samples, suggesting low sensitivity [56]. In contrast, flow cytometry has been validated in disseminated RMS, showing a higher specificity than PCR for CTC detection [69]. Taken together, these findings suggest that CTC detection in a specific sarcoma subtype may depend on the technique used for isolation and detection. Importantly, we note that standardized performance metrics across detection methodologies have not been consistently evaluated in the current literature, limiting the ability to systematically compare them by marker dependence, subtype suitability, and clinical maturity, and highlighting the need for further investigation in this area. Furthermore, CTCs have been shown to circulate in the bloodstream prior to the development of overt metastasis, making them important tumor biomarkers of early dissemination [70]. Selecting the optimal technique for their isolation and detection is pivotal, especially as enhanced technologies are currently under investigation. In particular, a protocol based on a microfluidic chip-type cell sorter demonstrated the detection of CTCs from a peripheral blood sample of a patient with locally advanced myxofibrosarcoma [62]. These findings suggest that CTC detection may warrant further investigation as a potential adjunct for disease surveillance, particularly in non-metastatic settings.

## 4. Clinical Implications of CTCs

In precision oncology, CTCs have emerged as promising investigational biomarkers for monitoring disease progression, assessing treatment response, and determining risk stratification. Their use has been well established in epithelial-derived malignancies, as the presence of epithelial cell markers facilitates their detection. In contrast, sarcomas comprise a highly heterogeneous group of mesenchymal-derived malignancies that typically lack common molecular features that can be used for CTC detection. Moreover, although their primary route of metastatic dissemination is hematogenous, the isolation of CTCs from the circulation is technically demanding, primarily due to the lack of standardized protocols for their detection, coupled with their scarcity in the bloodstream. Thus, their clinical utility remains largely under investigation. In the following paragraphs, we summarize the current evidence on the potential clinical and prognostic value of CTCs in STS.

### 4.1. CTC Isolation Across Sarcoma Subtypes

As briefly described, STS can be broadly classified into TRSs and CKSs [71]. Traditionally, CTCs were theorized to be particularly useful in TRSs compared with CKSs, owing to the presence of recurrent fusion transcripts that could be used for targeting [72]. Nevertheless, current findings suggest that CTC detection may be feasible in both TRSs and CKSs. A pilot study demonstrated the presence of CTCs in myxoid liposarcoma, a sarcoma subtype classically associated with the *FUS–DDIT3* fusion transcript. Likewise, CTCs were detected in angiosarcoma, leiomyosarcoma, myxofibrosarcoma, and undifferentiated pleomorphic sarcoma, all of which are commonly recognized as CKSs [63]. Collectively, these findings challenge the notion that CTC detection is limited to TRSs, suggesting that their detection may also be feasible across different sarcoma subtypes, including CKSs. An overview of published studies evaluating the use of CTCs in STS, along with the techniques used for their detection, is summarized in Table 1.

### 4.2. Clinical Implications in Treatment Response Monitoring and Survival

The clinical utility of CTCs as potential dynamic biomarkers for monitoring treatment response and establishing prognosis is currently under investigation. In a phase Ib study of patients with potentially resectable STS receiving olaratumab monotherapy followed by olaratumab plus doxorubicin, CTC enumeration using a validated fluorescence-based scanning method demonstrated dynamic changes that appear to correlate with therapeutic efficacy [74]. An initial rise in CTC counts was observed in cycle 1 day 8, followed by a significant reduction by cycle 3 day 1. Of note, the decrease in CTC counts after olaratumab monotherapy was substantially greater in patients achieving disease control compared with those without disease control [74]. Similarly, CTCs detected at diagnosis in patients with high-grade sarcoma declined after successful treatment and remained detectable in those without radiographic evidence of disease prior to the development of overt metastasis, further suggesting that CTC detection may reflect early tumor dissemination [59].

In this context, evidence supporting the use of CTCs as biomarkers of MRD and disease recurrence risk stratification has been reported in other solid tumors. CTC detection in patients without evidence of overt metastatic disease has been found to be associated with reduced disease-free survival, including both locoregional and distant recurrence [76,77]. Experimental studies have also suggested that local invasion and intravasation of cancer cells can occur quickly, even before a clinically evident malignancy can be detected [70]. Despite these findings, a consistent and clinically actionable “lead time” between CTC detection and radiological recurrence has not been established. Current evidence in STS remains insufficient to guide disease monitoring, which further underscores the need for prospective longitudinal studies to determine whether CTC monitoring can define a clinically meaningful window for intervention. Furthermore, although CTCs may reflect residual disease or early dissemination, a positive CTC count alone is insufficient to unequivocally predict recurrence or metastatic progression and, therefore, CTC positivity should be interpreted cautiously.

Beyond treatment-response monitoring and disease progression assessment, CTCs may also carry potential prognostic value in STS. It has been found that cell-surface vimentin-positive CTCs detected in pediatric and young adult sarcoma patients are correlated with significantly worse overall survival compared with CTC-negative patients [75]. Moreover, CTC characterization, beyond enumeration, may also provide prognostic value. It has been shown that high aneuploidy scores are associated with worse overall and progression-free survival in patients with metastatic STS, in whom aneuploid CTCs were detected using fluorescence in situ hybridization [73]. Collectively, these findings suggest that CTCs may serve as dynamic biomarkers for treatment-response monitoring and as potential adjuncts in risk stratification frameworks, although their clinical implementation in STS requires validation in larger prospective cohorts.

#### Comparison with Other Liquid Biopsy Approaches

Alternative liquid biopsy approaches, including cfDNA and its tumor-derived fraction (ctDNA), have gained increasing recognition, with some findings aligning with those observed using CTCs. Early decline in ctDNA has been shown to correlate with radiographic response and improved survival in patients with STS undergoing chemotherapy in the neoadjuvant or unresectable/metastatic setting [78]. Likewise, detection of ctDNA before treatment and its persistence following chemotherapy was significantly linked to poorer overall survival and objective response rates in patients with advanced leiomyosarcoma [79]. Similarly, plasma ctDNA tumor fraction was found to be significantly correlated with shorter progression-free and overall survival in patients with advanced sarcoma, and multivariate analysis identified this tumor biomarker as an independent predictor of survival [80]. Similar trends were made for CTCs in STS patients treated with olaratumab plus doxorubicin, where the reduction in CTCs was significantly larger in patients who achieved local disease control [74]. In addition to treatment-response monitoring and prognostication, ctDNA has been detected in patients with STS before the appearance of radiographic recurrence, and CTC detection has also been explored as a potential early indicator of disease evolution [62,81].

Although both CTCs and ctDNA provide unique advantages over conventional imaging and tumor biopsy, important distinctions and limitations between these liquid biopsy approaches should be considered [12]. The study of CTCs as intact and viable tumor cells enables their phenotypic, functional, and genomic characterization at the single-cell level, including the evaluation of EMT states, tumor cell plasticity, and potential therapeutic targets. In contrast, ctDNA provides a highly sensitive and quantitative assessment of tumor genomic burden, offering comprehensive genetic and epigenetic information, while facilitating the detection of tumor-associated alterations [12]. However, these advantages must be interpreted in light of important limitations. The utility of CTCs is strongly influenced by tumor heterogeneity and their analysis is further complicated by the low quantity of these cells in the bloodstream. Moreover, single-cell CTC analyses remain technically challenging, with limited standardization across platforms and variable reproducibility, which currently restricts their widespread clinical implementation [12,51]. By contrast, ctDNA is a fragmented biomarker, providing limited insight beyond genomic alterations and remaining susceptible to signal dilution by the presence of cfDNA from non-tumor sources. In addition, ctDNA reflects genetic alterations present in dying tumor cells and may not accurately represent the biologically active tumor cell population [12]. Collectively, although comparative studies between these approaches in STS are limited, the outlined advantages and limitations suggest that CTCs and ctDNA may represent complementary approaches.

### 4.3. Ongoing and Completed Clinical Trials

Several ongoing clinical trials are currently evaluating the role of CTCs in STS. The key details of these studies are summarized in Table 2.

## 5. Conclusions and Future Directions

CTCs represent a unique tumor-derived material that is expanding the understanding of tumor biology and hold promise as biomarkers for treatment-response monitoring and prognostication. Nevertheless, their use as clinically valuable tools in STS faces significant challenges. For instance, STS histological and molecular heterogeneity represents a significant limitation for CTC detection, isolation, characterization, and clinical interpretation, since common molecular features are typically absent in mesenchymal-derived malignancies and findings are unlikely to be universally applicable across sarcoma subtypes. In this context, stem cell-like phenotypes in CTCs represent a promising feature for future characterization, as, according to the CSC theory, common biological features may exist that could be used for targeting. Furthermore, their scarcity in the bloodstream, coupled with the absence of standardized laboratory protocols, constitutes a major limitation for the clinical adoption of CTCs. To overcome these technical challenges, emerging technologies for their sorting, isolation, and detection are currently under investigation. In sum, future research should focus on developing subtype-specific CTC detection methodologies and on achieving a deeper understanding of the CSC phenotype, as the optimal CTC detection approach likely varies across sarcoma subtypes. Additionally, attention should be given to the standardization of laboratory platforms to improve reproducibility and facilitate the integration of CTC analysis into routine clinical practice.

## Figures and Tables

**Table 1:** Summary of Published Studies on Circulating Tumor Cells in Soft Tissue Sarcomas.

Author	Year	STS Subtype	Detection Method	Target Marker	Key Findings
Kelly et al. [56]	1996	ARMS	RT-PCR	*PAX3–FKHR*; *PAX7–FKHR*	*PAX3–FKHR* was detected in bone marrow, including 2 histology-negative samples; fusion transcripts were not detected in peripheral blood.
Hoshino et al. [52]	2009	ASPS	RT-PCR	*ASPSCR1-TFE3*	*ASPSCR1–TFE3* was detected in peripheral blood from an ASPS patient with distant metastases; nested RT-PCR detected 50 tumor cells/2 mL of blood.
Almazán-Moga et al. [69]	2014	RMS	Flow cytometry	PAX3	PAX3 was expressed in all RMS tumors analyzed, and flow cytometry detected PAX3-positive/CD45-negative RMS cells in peripheral blood spiking experiments.
Domingos Chinen et al. [57]	2014	DDLPS; ES; SS *	Immunocytochemistry (ISET)	vimentin; pan-cytokeratin; CD45^−^	CTCs were detected in all patients with high-grade and metastatic sarcomas, with numbers ranging from 2 to 48 per 8 mL of blood.
Hayashi et al. [59]	2017	ARMS; DDLPS; ERMS; SS *	Immunofluorescence	vimentin; CD45^−^	CTCs were detected in 16/18 newly diagnosed patients and in 3/9 patients with no radiographic evidence of disease, all of whom (3/3) relapsed within 1–2 months.
Mihály et al. [54]	2018	SS	RT-PCR	*SS18–SSX1*; *SS18–SSX2*	*SS18–SSX2* fusion transcript was detected by ddPCR in 1/15 patients; nested PCR did not detect fusion transcripts in peripheral blood.
Hasegawa et al. [62]	2019	MFS	Microfluidic cell sorting (On-chip Sort)	*KMT2B*	One CTC was detected 3 months after surgery in a locally advanced MFS patient, and the primary tumor *KMT2B* mutation was confirmed in the CTC.
Przybyl et al. [55]	2019	SS	RT-PCR	*SS18–SSX1*; *SS18–SSX2*	*SS18–SSX1* fusion transcript was detected in peripheral blood from 2/38 SS patients; both had localized disease at blood collection.
Napolitano et al. [73]	2020	DDLPS; LMS; UPS; other STS	Fluorescence in situ hybridization	CEP3; CEP7; CEP8; CEP17; CEP20; *p16*; *hTERT*	Aneuploid CTCs were detected in 4/4 metastatic STS patients; high aneuploidy score was associated with shorter progression-free and overall survival.
Martín-Broto et al. [74]	2021	LPS; other STS	Immunofluorescence	cytokeratin; CD45^−^	CTC counts decreased after olaratumab monotherapy in 11/19 patients with disease control and 5/16 patients without disease control.
Dao et al. [75]	2021	RMS; other STS *	Immunofluorescence	vimentin; CD45^−^	CSV^+^ CTCs were detected more frequently in patients with active sarcoma than in long-term survivors; CSV^+^ CTC positivity was associated with poorer overall survival.
Young et al. [63]	2024	AS; DDLPS; LMS; MFS; MLPS; UPS	Immunofluorescence	vimentin; pan-cytokeratin; CD45^−^	CTCs were detected in all 13 STS patients; total CTC number was associated with primary tumor size but not with localized versus advanced disease.

ARMS: alveolar rhabdomyosarcoma; AS: angiosarcoma; ASPS: alveolar soft part sarcoma; CD45: cluster of differentiation 45; CEP: chromosome enumeration probe; CSV: cell surface vimentin; CTCs: circulating tumor cells; DDLPS: dedifferentiated liposarcoma; ddPCR: droplet digital polymerase chain reaction; ERMS: embryonal rhabdomyosarcoma; ES: epithelioid sarcoma; *hTERT*: human telomerase reverse transcriptase; ISET: isolation by size of epithelial tumor cells; LMS: leiomyosarcoma; LPS: liposarcoma; MFS: myxofibrosarcoma; MLPS: myxoid liposarcoma; RMS: rhabdomyosarcoma; RT-PCR: reverse transcription polymerase chain reaction; SS: synovial sarcoma; STS: soft tissue sarcoma; UPS: undifferentiated pleomorphic sarcoma. * This study included a mixed sarcoma population comprising both soft tissue and bone sarcomas; only STS subtypes are listed in this table.

**Table 2:** Clinical Trials Assessing Circulating Tumor Cells Registered at ClinicalTrials.gov (accessed on 5 May 2026).

Clinical Trial ID	Study Title	Study Type	Study Population	Sample Size	Aim	Role of CTC Assessment *
Clinical Trials Evaluating CTCs in STS						
NCT07401355	Improved Management of Soft Tissue Sarcomas Patients With an Optimized and Innovative Sorting Technology for Circulating Tumor Cells	I	Metastatic or unresectable locally advanced STS	100	To develop a method for detecting and sorting CTCs from liquid biopsies in patients with metastatic or locally advanced STS.	CTC-based primary study objective
NCT02983539	Detection of Circulating Tumor Cells in Patients With Metastatic Sarcomas	O	Metastatic STS (LMS, UPS, SS, LPS)	20	To isolate and quantify CTCs from peripheral blood samples obtained from patients with different metastatic STS subtypes.	CTC-based primary study objective
NCT05427461	Circulating “Cancer Cells/Macrophage” Hybrid Cells in Patients With Sarcoma, Part 2	I	Localized or metastatic LMS	20	To longitudinally evaluate CTCs and cancer cell–macrophage hybrid cells in peripheral blood samples among patients with localized or metastatic LMS.	CTC-based primary study objective
Clinical Trials in Mixed Sarcoma Populations, Including Bone and STS						
NCT02849366	Combination of Cryosurgery and NK Immunotherapy for Recurrent Sarcoma	I	Recurrent sarcoma	30	To assess the safety and efficacy of cryotherapy plus NK immunotherapy in recurrent sarcoma.	Supportive biomarker assessment
NCT04512495	Circulating “Cancer Cells/Macrophage” Hybrid Cells in Patients With Sarcoma?	I	Bone and STS subtypes (LMS, DDLPS, MLPS, SS, OS, EWS)	60	To evaluate the rate of patients with circulating “cancer cell/macrophage” hybrid cells in the peripheral blood.	CTC-based primary study objective
NCT03357315	Safety and Efficacy Study of Mix Vaccine in Sarcoma Patient	I	Metastatic sarcoma	30	To determine the safety and efficacy of mix vaccine in small metastases of sarcoma.	Supportive biomarker assessment
Clinical Trials in Mixed Tumor Populations, Including Mesenchymal and Epithelial Malignancies						
NCT04239443	Peripheral CTC Detection and CTC-based PD-L1 Antibody Immunofluorescence Detection to PD-1 Monoclonal Antibody SHR-1210 and Apatinib in Second-line and Back-line Treatment of Advanced NSCLC, Soft Tissue Sarcoma, Uterine Cancer Clinical Research	I	Multiple cancers (NSCLC, STS, uterine cancer)	120	To evaluate the efficacy and safety of SHR-1210 plus apatinib in patients with advanced NSCLC, STS, and uterine cancer, while assessing baseline and dynamic CTC/CTC PD-L1 levels in relation to treatment response and prognosis.	Supportive biomarker assessment
NCT04935333	Prospective, Observational and Multicenter Case-control Study to Evaluate the Precision of the Preoperative Molecular Diagnosis of Uterine Tumors by Liquid Biopsy	O	Myometrial tumor (leiomyoma or leiomyosarcoma)	600	To evaluate the diagnostic performance of liquid biopsy based molecular detection of CTCs and other tumor-derived biomarkers for the preoperative diagnosis of uterine tumors.	CTC-based primary study objective
NCT06967961	Research of Double-positive Circulating Cells (Tumor Marker/CD45^+^) in Several Types of Metastatic Cancers	I	Multiple cancers (including STS)	450	To determine the proportion of patients with detectable circulating double-positive tumor cells in peripheral blood using flow cytometry or microfluidic circulating-cell enrichment/detection platforms.	CTC-based primary study objective
NCT04628806	Heat Shock Protein 70 to Quantify and Characterize Circulating Tumor Cells in Patients With Advanced or Metastatic Tumors	O	Metastatic cancers (including sarcoma)	120	To evaluate whether CTC counts detected using HSP70- and EpCAM-based assays are associated with radiographic treatment response.	Supportive biomarker assessment

CTCs: circulating tumor cells; CD45: cluster of differentiation 45; DDLPS: dedifferentiated liposarcoma; EpCAM: epithelial cell adhesion molecule; EWS: Ewing sarcoma; HSP70: heat shock protein 70; I: interventional; LMS: leiomyosarcoma; LPS: liposarcoma; MLPS: myxoid liposarcoma; NK: natural killer cells; NSCLC: non-small cell lung cancer; O: observational; OS: osteosarcoma; PD-1: programmed cell death protein 1; PD-L1: programmed death-ligand 1; SHR-1210: camrelizumab (anti–PD-1 monoclonal antibody); SS: synovial sarcoma; STS: soft tissue sarcoma; UPS: undifferentiated pleomorphic sarcoma. * Role of CTC Assessment was classified as CTC-based primary study objective when CTC or CTC-related biomarker detection, quantification, or characterization represented the primary objective of the study, and as supportive biomarker assessment when CTCs were evaluated as ancillary biomarkers within studies primarily assessing treatment response, disease monitoring, or prognostication.

## Data Availability

No new data were created or analyzed in this study. Data sharing is not applicable to this article.

## Abbreviations

The following abbreviations are used in this manuscript:

STS	Soft tissue sarcomas
TRSs	Translocation-related sarcomas
CKSs	Complex karyotype sarcomas
CTC/CTCs	Circulating tumor cells
cfDNA	Cell-free DNA
ctDNA	Circulating tumor DNA
EMT	Epithelial–to-mesenchymal transition
TME	Tumor microenvironment
DTCs	Disseminated tumor cells
CSC/CSCs	Cancer stem cells
MET	Mesenchymal-to-epithelial transition
RMS	Rhabdomyosarcoma
SS	Synovial sarcoma
MMP-14	Matrix metalloproteinase-14
EGFR	Epidermal growth factor receptor
ARGs	Anoikis-related genes
CFLAR	CASP8 and FADD-like apoptosis regulator
RT-PCR	Reverse transcription polymerase chain reaction
ddPCR	Droplet digital polymerase chain reaction
ISET	Isolation by size of epithelial tumor cells
cDNA	Complementary DNA
RBCs	Red blood cells
MRD	Minimal residual disease
ARMS	Alveolar rhabdomyosarcoma
AS	Angiosarcoma
ASPS	Alveolar soft part sarcoma
CD45	Cluster of differentiation 45
CEP	Chromosome enumeration probe
CSV	Cell surface vimentin
DDLPS	Dedifferentiated liposarcoma
ERMS	Embryonal rhabdomyosarcoma
ES	Epithelioid sarcoma
hTERT	Human telomerase reverse transcriptase
LMS	Leiomyosarcoma
LPS	Liposarcoma
MFS	Myxofibrosarcoma
MLPS	Myxoid liposarcoma
UPS	Undifferentiated pleomorphic sarcoma
EpCAM	Epithelial cell adhesion molecule
EWS	Ewing sarcoma
HSP70	Heat shock protein 70
I	Interventional
NK	Natural killer
NSCLC	Non-small cell lung cancer
O	Observational
OS	Osteosarcoma
PD-1	Programmed cell death protein 1
PD-L1	Programmed death-ligand 1
SHR-1210	Camrelizumab
